# Combining the Fecal Immunochemical Test with a Logistic Regression Model for Screening Colorectal Neoplasia

**DOI:** 10.3389/fphar.2021.635481

**Published:** 2021-03-17

**Authors:** Feiyuan Liu, Qiaoyun Long, Hui He, Shaowei Dong, Li Zhao, Chang Zou, Weiqing Wu

**Affiliations:** ^1^ Department of Scientific Research, The First Affiliated Hospital, Southern University of Science and Technology, Shenzhen People’s Hospital, Shenzhen, China; ^2^ Department of Clinical Research Center, The First Affiliated Hospital, Southern University of Science and Technology, Shenzhen People’s Hospital, Shenzhen, China; ^3^ Shenzhen Public Service Platform on Tumor Precision Medicine and Molecular Diagnosis, Shenzhen, China; ^4^ Department of Health Management, The First Affiliated Hospital, Southern University of Science and Technology, Shenzhen People’s Hospital, Shenzhen, China

**Keywords:** fecal immunochemical test, colorectal neoplasia screening, logistic regression model, funnel strategy, classifier model

## Abstract

**Background:** The fecal immunochemical test (FIT) is a widely used strategy for colorectal cancer (CRC) screening with moderate sensitivity. To further increase the sensitivity of FIT in identifying colorectal neoplasia, in this study, we established a classifier model by combining FIT result and other demographic and clinical features.

**Methods:** A total of 4,477 participants were examined with FIT and those who tested positive (over 100 ng/ml) were followed up by a colonoscopy examination. Demographic and clinical information of participants including four domains (basic information, clinical history, diet habits and life styles) that consist of 15 features were retrieved from questionnaire surveys. A mean decrease accuracy (MDA) score was used to select features that are mostly related to CRC. Five different algorithms including logistic regression (LR), classification and regression tree (CART), support vector machine (SVM), artificial neural network (ANN) and random forest (RF) were used to generate a classifier model, through a 10X cross validation process. Area under curve (AUC) and normalized mean squared error (NMSE) were used in the evaluation of the performance of the model.

**Results:** The top six features that are mostly related to CRC include age, gender, history of intestinal adenoma or polyposis, smoking history, gastrointestinal discomfort symptom and fruit eating habit were selected. LR algorithm was used in the generation of the model. An AUC score of 0.92 and an NMSE score of 0.076 were obtained by the final classifier model in separating normal individuals from participants with colorectal neoplasia.

**Conclusion:** Our results provide a new “Funnel” strategy in colorectal neoplasia screening via adding a classifier model filtering step between FIT and colonoscopy examination. This strategy minimizes the need of colonoscopy examination while increases the sensitivity of FIT-based CRC screening.

## Introduction

Colorectal cancer (CRC) is the fourth most common cancer, and accounts for around 10% of the newly diagnosed cases of cancers (Siegel et al., 2020). In 2019, CRC caused approximately 900,000 deaths worldwide (Dekker et al., 2019). CRC screening is a process of detecting adenomatous polyps or early cancerous change that are highly treatable (Atkin et al., 2010; Schoen et al., 2012) and is currently one of the most realistic approaches that reduce CRC-related mortalities (Oort et al., 2010).

Three main types of CRC screening strategies have been suggested by various international guidelines, which are physical-based, blood-based and faecal-based methods. Among them, physical-based methods such as colonoscopy are currently the most sensitive tests in CRC screening. However, due to its invasiveness and complexity, colonoscopy may not be acceptable as a population-based screening test (Gupta et al., 2013). Blood-based screening methods or liquid biopsies are a type of non-invasive screening methods which detect biomarkers in a patient blood sample (Hauptman and Glavač, 2017). Currently some available and innovative (published but not yet commercially available) blood-based CRC screening strategies include carcinoembryonic antigen (CEA) (Locker et al., 2006), carbohydrate antigen 19-9 (CA 19-9) (Kim et al., 2017), circulating tumor cells (CTCs) (Baek et al., 2019), cell-free DNA (cfDNA) (Vymetalkova et al., 2018), microsatellite instability (MSI) (Zeinalian et al., 2018), aberrant DNA methylation (*SEPT9* gene methylation status) (Warren et al., 2011), mRNAs (*ANXA3, CLEC4D, LMNB1, PRRG4, TNFAIP6, VNN1* and *IL2RB*) from peripheral blood (Marshall et al., 2010), microRNAs (miR-601, miR-760, miR-15b, miR-19a, miR-19b, miR-29a, miR-335) (Wang et al., 2012; Ahmed et al., 2013; Giráldez et al., 2013; Kanaan et al., 2013) and long noncoding RNAs (LncRNAs CRNDE-h, CCAT, HOTAIR) (Graham et al., 2011; Zhao et al., 2015). Feacal-based methods detect biomarkers in patients’ stool samples including guaiac-based faecal occult blood test (gFOBT), fecal immunochemical test (FIT) and multitargeted stool DNA test (FIT-DNA). Of these, gFOBT uses chemical guaiac to detect blood in stool. Due to its high false positive and negative rate, it requires three home-based stool samples per test (Kościelniak-Merak et al., 2018); FIT-DNA detects altered DNA in stool sample, and is more expensive than the other two tests. FIT uses antibodies specific to hemoglobin, and has the ability to detect low level of bleeding in stool samples. In comparison with physical-based screening methods, FIT is a non-invasive test and can be done without dietary or medication restrictions; in comparison with blood-based screening methods, FIT is cheaper and faster in the report generation process while yielding fairly reliable results. Hence FIT is recommended as a population level screening strategy (Chiu et al., 2013).

FIT-based CRC screening has now been widely applied in many Asia and European countries (Chen et al., 2011; Stegeman et al., 2012). However, there are some limitations in this strategy, such as low sensitivity for identifying certain types of polyps and some false-positive cases. The reported FIT sensitivity ranged from 25% to 100% and the specificity usually exceeded 90%, as summarized by Lee et al. (Lee et al., 2014). To increase the sensitivity of FIT-based screening, in this study, we established a funnel strategy via adding a model filtering step between FIT and colonoscopy examination. This filtering model was established by a logistic regression analysis using FIT results and six other features, and an AUC score of 0.92 was reached in discriminating colorectal neoplasia participants from normal ones.

## Materials and Methods

### Data Collection

This study was conducted in the Early Cancer Screening Center (ECSC) of the Health Management Department in Shenzhen People’s hospital, Guangdong, China. A total of 4,477 participants were recruited from customers who came for physical examination in the period from March 2019 to June 2020. No specific inclusion or exclusion criteria was applied. The demographic characteristics from all participants including age, sex, BMI (Body Mass Index), clinical history, diet habits and life styles were collected through a questionnaire survey.

### Fecal Immunochemical Test and Colonoscopy

A FIT testing was performed in all these 4,477 participants using a fully automated fecal occult blood analyzer (OC-SENSOR io, EIKEN Chemical Co., Ltd., Japan), and a value of 100 ng/ml was used as a cut-off based on the manufacturer’s instructions (FIT positive: >100 ng/ml). For FIT positive participants, colonoscopy examination (CSE) was performed by gastroenterologists from the Department of Anorectal Surgery in Shenzhen People’s Hospital. The following situations were considered as colorectal neoplasia (CSE Positive): colorectal polyps, adenoma and colorectal cancer. The rest including inflammation were considered as CSE Negative.

### Data Pre-Processing

The demographic and clinical characteristics of participants were divided into four domains that consist of 15 variables. The information of all 4,477 participants was listed in Supplementary Table S1 and the summary information of 155 FIT positive participants who went through colonoscopy examination was listed in Table 1. A series of data conversion were performed to facilitate subsequent analysis. For “Age,” a z-score was performed to avoid overfitting; for “BMI,” value < 20 was defined as 0, 20≤value≤25 was defined as 1, and value > 25 was defined as 2; for “Gender”, male was defined as 0 and female was defined as 1; for binary variables in clinical history category including tumor, family tumor, IAP (Intestinal adenoma or polyposis) and GDS (Gastrointestinal discomfort, including symptoms such as abdominal pain or discomfort, increased defecation frequency, black stool, blood/pus/mucus in the stool, constipation) and in life style category including smoking, drinking and pressure (here the pressure is defined as living or working pressure, which is the subjective judgment of the participants regarding to their mental status including anxiety and depression), a “yes” was defined as 1 and a “no” was defined as 0; For diet habit category including fruits, vegie and meat and for life style category including sports, “< 3 times/week” was defined as 0 (rarely) and “≥ 3 times/week” was defined as 1 (regularly).

**TABLE 1:** Features of FIT positive participants and their correlation with colorectal neoplasia.

Category			Total	CSE positive	CSE negative	*p* value
Basic information	Gender	Male	96	61	35	0.0026
		Female	59	22	37	
	Age	>55	48	34	14	0.0062
		≤55	107	49	58	
	BMI	20–25	88	45	43	0.6
		Else	67	38	29	
Clinical history	Tumor	YES	2	0	2	0.42
		NO	153	83	70	
	Family tumor	YES	45	24	21	1
		NO	110	59	51	
	IAP	YES	17	16	1	0.00098
		NO	138	67	71	
	GDS	YES	56	25	31	0.13
		NO	99	58	41	
Diet habits	Fruits	Rarely	44	20	24	0.27
		Regularly	111	63	48	
	Vegie	Rarely	6	2	4	0.55
		Regularly	149	84	68	
	Meat	Rarely	12	6	6	1
		Regularly	143	77	66	
Life styles	Smoking	YES	54	37	17	0.01
		NO	101	46	55	
	Drinking	YES	37	19	18	0.91
		NO	118	64	54	
	Sports	Rarely	71	31	40	0.035
		Regularly	84	52	32	
	Pressure	YES	83	43	40	0.76
		NO	72	40	32	

### Feature Selection

After data pre-processing, the number of participants in each category was summarized and listed in Table 1. A chi-square test was used to explore the correlation between each feature and CSE results, and a *p* value of 0.05 was used as the cutoff for statistical significance.

**FIGURE 1:**
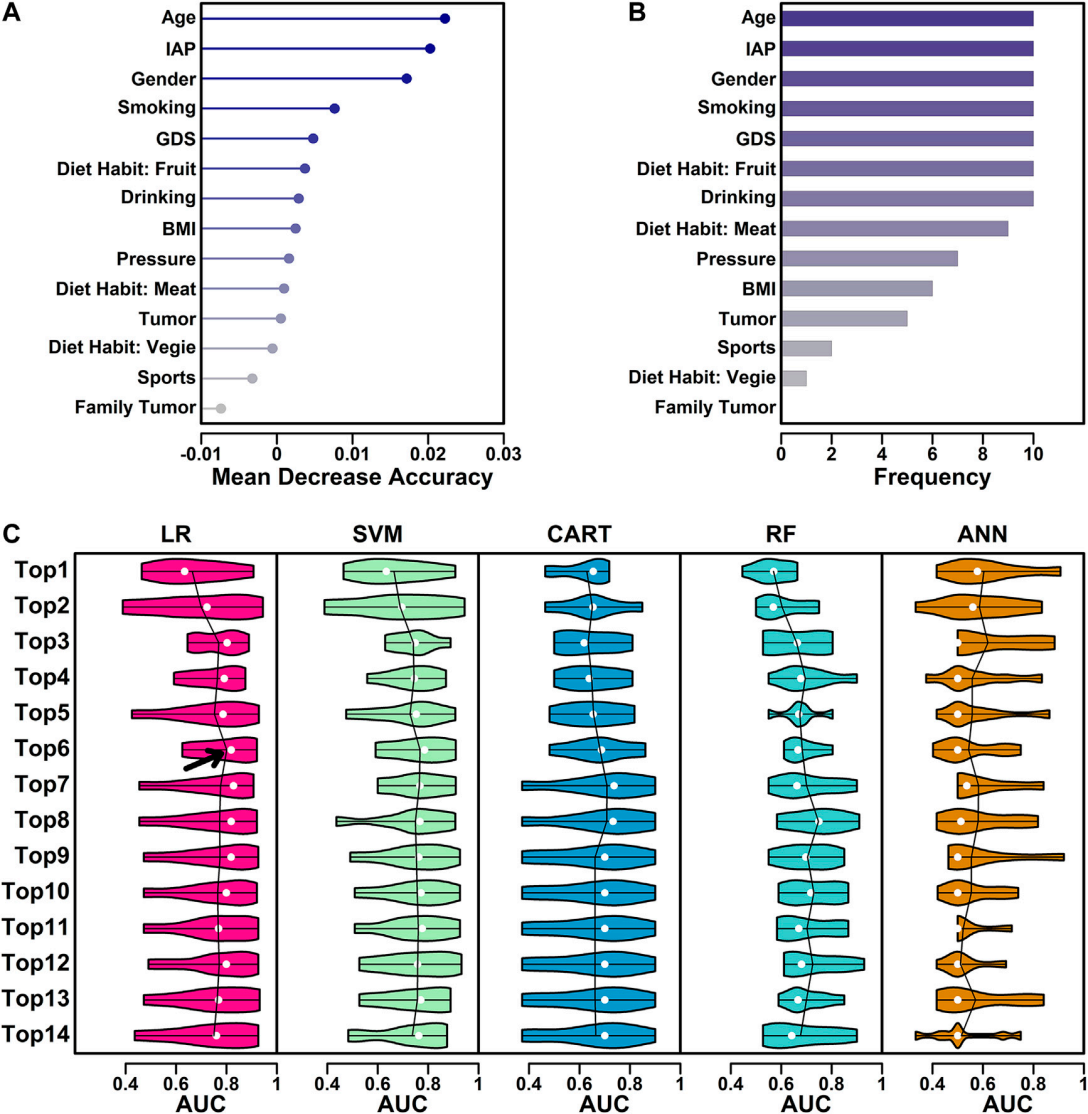
Feature selection process. **(A)** MDA values of all 15 features. **(B)** The number of the appearances in top 10 ranked variables in every iteration. **(C)** Performance of five different algorithms using different combinations of features. The AUC values from 10 iterations were illustrated as violin plot, with mean value highlighted in lines and median value highlighted in white dots.

To filter features for model building, a random forest process was performed and a Mean Decrease Accuracy (MDA) score was used to evaluate the feature contribution value. In this step, a 10-fold cross validation process was used and 10 iterations were performed. The average MDA scores of all 15 features were ranked and listed (Figure 1A), and the frequencies of the appearances of top 10 important features were also ranked and listed (Figure 1B).

### Model Selection

Five classical algorithms were used in the model selection step, including LR (Logistic regression), CART (Classification and regression tree), SVM (Support vector machine), ANN (Artificial neural network) and RF (Random forest). In this study, R function glm() was used to perform LR analysis with all parameters set as default except connection (set to “binomial”) (Dobson 1990); R function rpart() was used to perform CART analysis with all parameters set as default (Breiman et al., 1984); R function svm() was used to perform SVM analysis with the kernel parameter set as “linear” and scale parameter set as “FALSE” (Fan et al., 2005); R function nnet() was used to perform ANN analysis with the size parameter set as 1, maxit parameter set as 1,000 and entropy parameter set as “TRUE” (Ripley, 2008); R function randomForest() was used to perform RF analysis with the netree parameter set as iterative manner and optimal 67 selected (Breiman 2001). All the parameters were set based on the manuals of each R function.

For the features ranked based on their MDA scores, different combinations (top N) of features were tested in all five algorithms and their AUC (Area Under Curve) values in all 10 iterations were calculated using R function pROC() and illustrated as violin plots (Figure 1C). Average AUC was used to select the best algorithm + best feature combinations (top N).

### Model Evaluation

Six features including “Age,” “IAP,” “Gender,” “Smoking,” “GDS” and “Diet Habit: Fruit” were used in the final model generation. AUC score and NMSE (Normalized mean squared error) value were used in the evaluation of model performance. R package pROC was used in the calculation of AUC. The NMSE value was calculated using formula: mean((predicted value−observed value)^2)/mean((mean(observed value)−observed value) ^ 2).

### Ethical Considerations

This study was approved by the Ethical Committee Board of the Shenzhen People’s hospital. All the participants were provided with a cover letter containing information regarding the research purpose and methods. Written consents were obtained from all participants.

## Results

### Study Design

**FIGURE 2:**
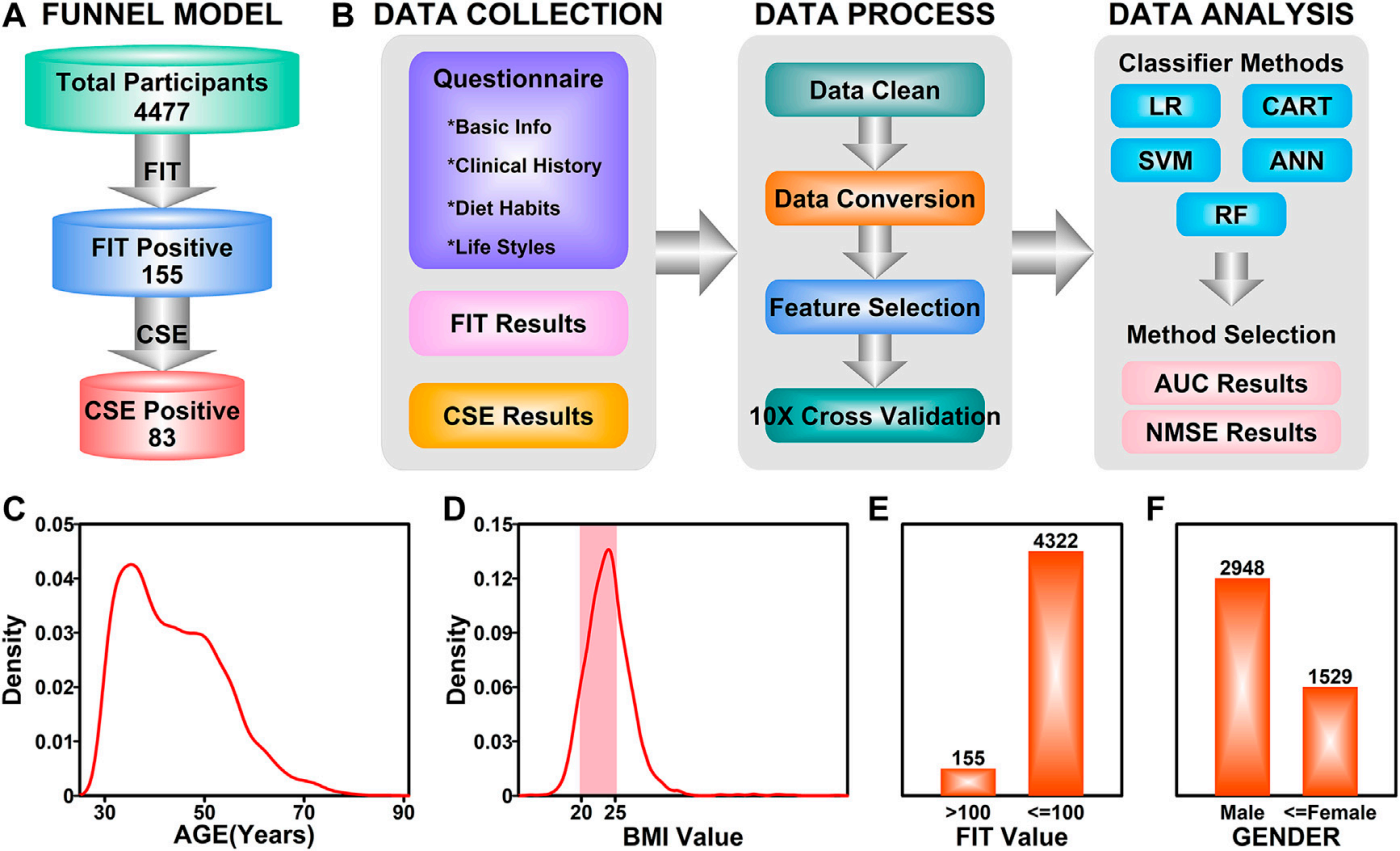
Study design. **(A)** An illustration of funnel model in colorectal screening. **(B)** A workflow describing the details in establishing the final classifier model. **(C)** Density distribution of ages among all participants. **(D)** Density distribution of BMI values among all participants. Highlighted area **(pink)** represents normal BMI. **(E)** Bar plot representing the ratio of FIT positive participants. **(F)** Bar plot representing the ratio of male and female participants.

The number of participants in each step were illustrated in Figure 2A. The aim of this study is to generate a classifier model to evaluate the likelihood of colorectal neoplasia based on FIT results and a cohort of other features. The workflow of this study was illustrated in Figure 2B. For the 155 FIT positive participants, the CSE results together with the information from questionnaires were collected. After data cleaning and data conversion steps, all the features were evaluated and the top ranked features were selected and used in the following 10-fold cross validation process. Five analytical methods including LR, SVM, CART, ANN and RF were used in the data analysis step, and the AUC and NMSE scores were used in judging the performance of the classifier model.

### Characteristics of Participants

These 4,477 participants have the age ranging from 30 to 86 years (Figure 2A). The age distribution of the participants was illustrated in Figure 2C, with a peak in 35 years. The BMI value distribution of the participants was illustrated in Figure 2D, with a majority of values (56.7%, pink field) falling in the 20–25 (20 ≤ BMI ≤ 25 is defined as normal) normal range. Among these 4,477 participants, 155 of them have a FIT score over 100 ng/ml (Positive), while the rest of the participants were FIT negative, as illustrated in Figure 2E; 2,948 of the participants were males (65.8%) and 1,529 of them were females (34.2%). After colonoscopy examination of the 155 FIT positive participants, 83 of them (53.5%) were diagnosed with colorectal neoplasia (CSE Positive).

### Features of Fecal Immunochemical Test Positive Participants

**FIGURE 3:**
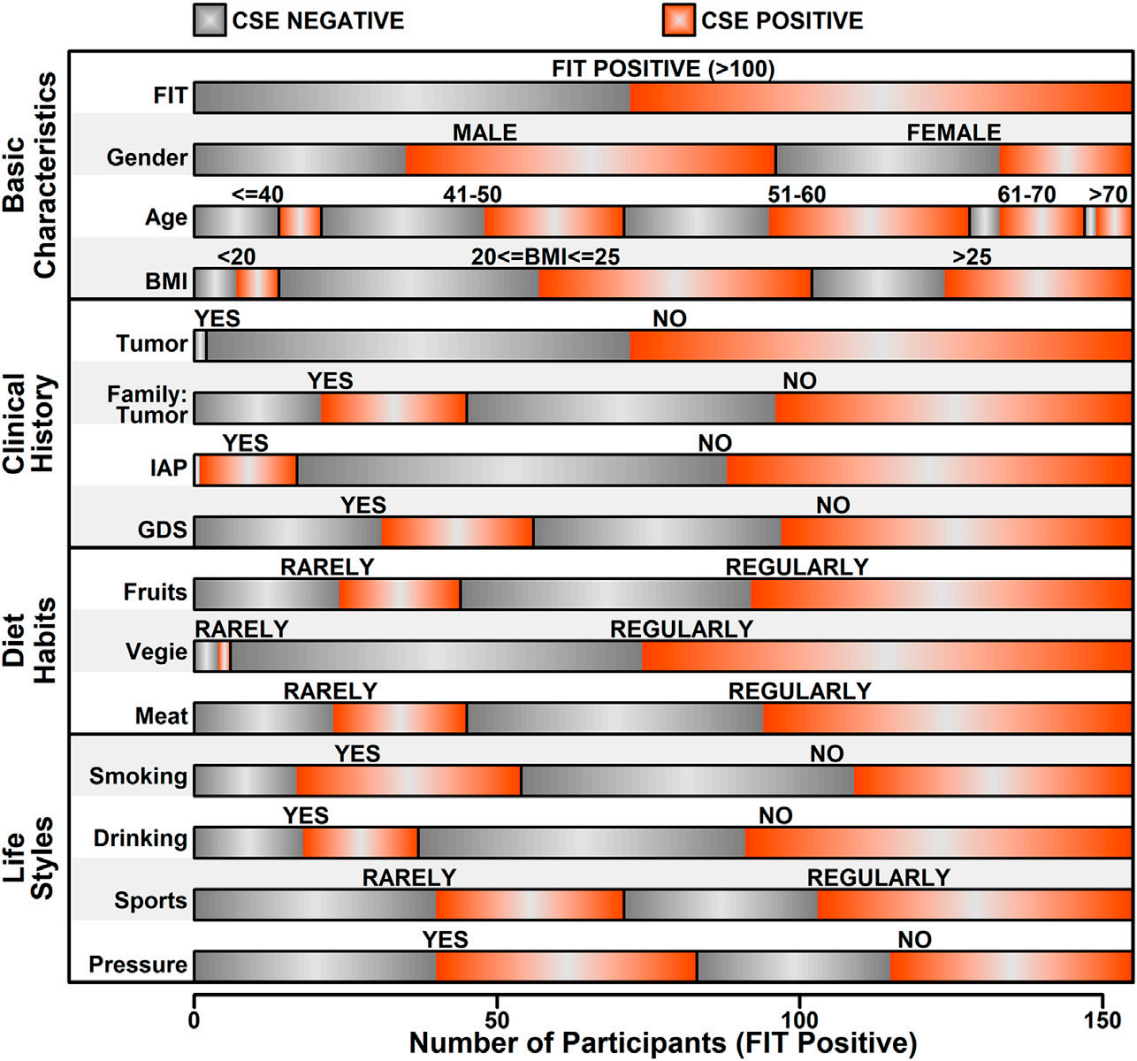
Summary of the features of FIT positive participants.

In this study, after data preprocess, only features with relatively high data integrity were selected, which yields 15 features, as listed in Supplementary Table S1. Information relating to these 15 features was further extracted from the raw data of 155 FIT positive participants, and the details of these features were illustrated in Figure 3. Among these 155 FIT positive participants, 83 of them were CSE positive (53.5%), and 72 were CSE negative (46.5%). These features were further divided into four categories including “Basic information,” “Clinical History,” “Diet Habits” and “Life Styles.”

Among all the 15 features, five of them showed a significant correlation with colorectal abnormal symptoms (P value <0.05, as listed in Table 1), which are “Gender,” “Age,” “IAP,” “Smoking” and “Sports.” Regarding to “Gender,” among 96 FIT positive male participants, 61 of them were diagnosed as CSE positive (63.5%); among 59 FIT positive female participants, 22 of them were diagnosed as CSE positive (37.3%), indicating a higher incidence of colorectal neoplasia in FIT positive males (*p* value = 0.0026). Regarding to “Age,” 48 of FIT positive participants were over 55 years old, with 34 of them diagnosed as CSE positive (70.8%); 107 of FIT positive participants were less than 55 years old, with 49 of them diagnosed as CSE positive (45.8%), indicating a higher incidence of colorectal neoplasia in older FIT positive participants (*p* value = 0.0062). Regarding to the “IAP,” 17 of the FIT positive participants had a history of intestinal adenoma or polyps, in which 16 of them were diagnosed as CSE positive (94.1%); for the rest of the FIT positive participants with no history of intestinal adenoma or polyps, 67 of them were diagnosed as CSE positive (48.6%), indicating a higher incidence of colorectal neoplasia in FIT positive participants with a history of intestinal adenoma or polyps (*p* value = 0.00098). Regarding to “Smoking,” 54 of the FIT positive participants had a smoking history, in which 37 of them were diagnosed as CSE positive (68.5%); for the rest of FIT positive participants with no smoking habits, 45 of them were diagnosed as CSE positive (44.6%), indicating a higher incidence of colorectal neoplasia in smoking FIT positive participants (*p* value = 0.010). Regarding to “Sports,” 71 of the FIT positive participants rarely played sports (less than two times/week), in which 31 of them were diagnosed as CSE positive (43.7%); for the rest of FIT positive participants who regularly played sports (more than two times/week), 52 of them were diagnosed as CSE positive (61.9%), indicating a higher incidence of colorectal neoplasia in FIT participants playing sports regularly (*p* value = 0.035).

### Feature Selection

These 15 features were further screened based on the contributions to the final models. In this step, a random forest process was used in the screening, and a 10-fold cross-validation process was used to eliminate the difference caused by sample randomness. These 15 features were first ranked based on the average MDA scores from 10 iterations, and the results were listed in Figure 1A. These 15 features were further ranked by the number of the appearances in top 10 ranked variables in every iteration, and the results were listed in Figure 1B.

The feature selection process was further performed using five classical algorithms including logistic regression (LR), support-vector machine (SVM), classification and regression tree (CART), random forest (RF) and artificial neural network (ANN). LR utilizes the logistic function to estimate a binary dependent variable, which is, in this study, CSE positive or CSE negative (Tolles and Meurer, 2016); SVM uses a set of training examples to build an algorithm which assigns new examples to one of the two categories (positive/negative) (Cortes and Vapnik, 1995); CART utilizes a predictive model (decision tree) to generate a conclusion (tree leaves, positive/negative) based on the observations (tree branches, training sets) (Barlin et al., 2013); RF is an ensemble method combining multiple learning algorithms such as classification and regression, to output the class of individual trees (positive/negative) based on a multitude of decision trees constructed during training (Breiman, 2001). ANN is based on an artificial neural network constructed by neurons (nodes) and connections (edges). During a training process, a probability-weighted association was generated between input (different characteristics) and result (CSE results) (Renganathan, 2019). These five machine learning methods are currently most widely used algorithms, and they were all included in this study. Their performance was summarized in Figure 1. The ranked features from Figure 1A was used as the input, and the AUC scores were used as the judgements. The performance was summarized and illustrated in Figure 1C. Among all the five algorithms and different combinations of features, the LR algorithm with top six features yielded the highest mean AUC value (highlighted with arrow), indicating a combination of these six features has the best separating ability in discriminating CSE positive participants from CSE negative ones, hence these six features, “Age,” “IAP,” “Gender,” “Smoking,” “GDS” and “Diet Habit: Fruit” were chosen in the final model generation.

### Model Performance

**FIGURE 4:**
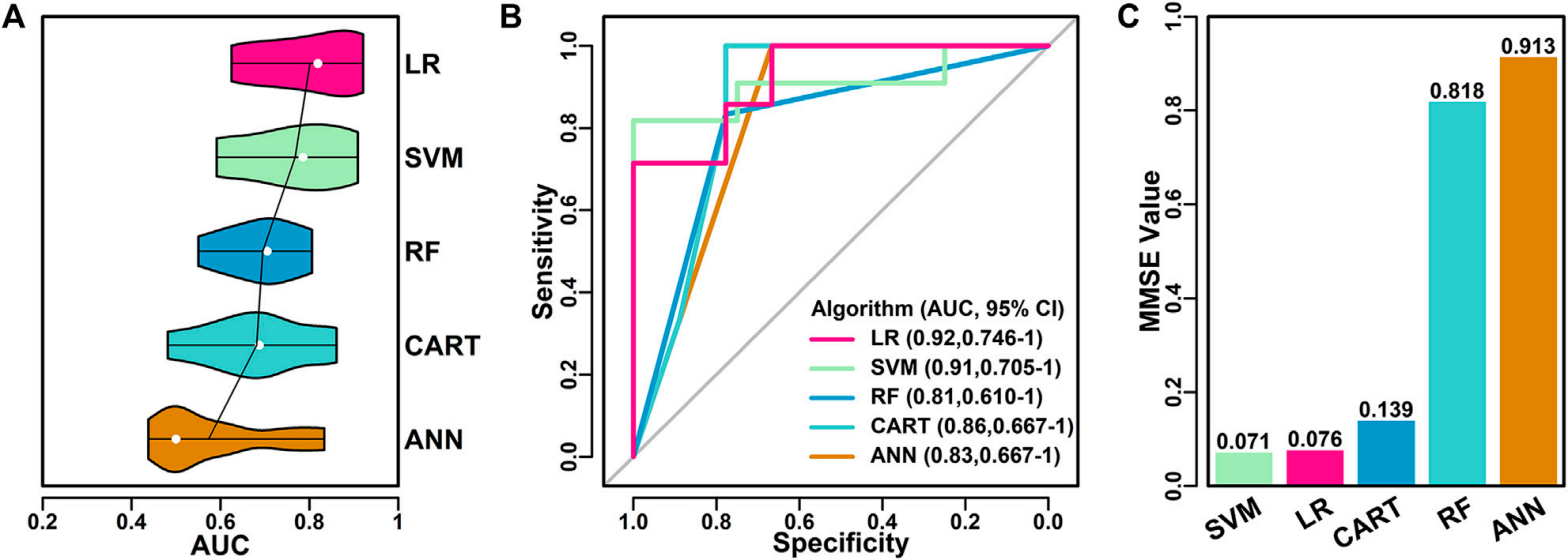
Performance of final classifier models. **(A)** Performance of five models using different algorithms with top six features as input. The mean AUC scores were highlighted in lines and the median AUC scores were highlighted in white dot. **(B)** ROC curves of different models. **(C)** NMSE values of different models.

The top six features were further applied in five algorithms for model building, and the performance of these models were evaluated and compared with each other. The AUC score distribution of each model was shown in Figure 4A. The highest, lowest, mean and median AUC values for each model were listed as follows:

LR (0.92, 0.63, 0.80, 0.82); SVM (0.91, 0.59, 0.77, 0.79); RF (0.81, 0.55, 0.69, 0.71); CART (0.86, 0.48, 0.68, 0.69); ANN (0.83, 0.44, 0.57, 0.50).

Among all five algorithms, LR model performed the best, with the highest AUC score of 0.92 in one of the 10 iterations. ROC curves of the best models using different algorithms were shown in Figure 4B. LR model has the highest AUC value (0.92), followed by SVM model (0.91), RF model (0.81), CART model (0.86) and ANN model (0.83).

The performance of the best models using different algorithms were also evaluated using the average NMSE value, as shown in Figure 4C. SVM and LR models have the lowest scores (0.071 and 0.076), followed by CART (0.139), RF (0.818) and ANN (0.913), which was in consistent with the results of AUC evaluations.

## Discussion

FIT has been recommended as a non-invasive strategy in CRC screening, and the sensitivity varies between 25 and 100% in many reports, as summarized by Lee et al. (2014). To further increase the sensitivity of FIT in identifying colorectal neoplasia, we combined FIT results with six other demographic and clinical characteristics, and established a LR classifier model, which yields an AUC of 0.92 in distinguishing colorectal neoplasia participants from false positive ones.

In this study, 83 FIT positive participants were diagnosed with colorectal neoplasia, with a sensitivity of 53.5%. The cut off value for FIT positive was set at 100 ng/ml, as suggested by the FIT equipment manufacturer. 100 ng/ml was widely used as FIT positive judgment standard (Chen et al., 2011; Crotta et al., 2012), and different cut off values do not have too much effects on final results, as discussed by Wilschut et al. (2011), in which FIT values ranging from 50 to 200 ng/ml were used as cutoffs, and the sensitivities varies a little around 60%, which is comparable to our sensitivity result. Multiple rounds of FITs might improve the screening results, as discussed in many studies (Crotta et al., 2012; Kapidzic et al., 2014; Jensen et al., 2016), however, in this study, due to the limitations of time and cost, only one round of FIT was performed to all participants. In the future, multiple rounds of FITs might help in generating a better and more accurate classifier model.

Six features were involved in establishing the final classifier model. Among them, Age, Gender, IAP and Smoking showed significant correlations with development of colorectal neoplasia (*p* value <0.05), as listed in Table 1. Regarding to age and sex, it has long been observed that these two factors are directly associated with the occurrence of colorectal cancer (Siegel et al., 2019; Siegel et al., 2020). Siegel et al. (2014) showed that the number of new CRC cases and deaths associated with CRC increases with age, and these numbers are higher in males in comparison with that in females, which is in consistent with our results. Regarding the IAP, the correlation between personal history of polyps or adenomas and colorectal cancer or neoplasia have also been reported. Stark et al. (2006) showed that colon polyp was a risk factor associated with CRC. Saini et al. (2006) showed that patients with a history of adenoma were more likely to have recurrent adenomas. As of smoking, there is consistent evidence of relationships between dose-responsive smoking and colorectal neoplasia Fagunwa et al. (2017) or CRC (Kozma et al., 2012; Akter et al., 2020), even in a passive smoking manner (Yang et al., 2016). Regarding to drinking, although there were studies reported the relationship between drinking and occurrence of CRC (Zisman et al., 2006; Fagunwa et al., 2017), the correlation between drinking and colorectal neoplasia is not significant in our study, which might be caused by the limited number of participants.

The relationship between sports (Physical activity) and CRC incidence is controversial in many studies. Papadimitriou et al. (2020) showed an inverse association between physical activity and incidence of colorectal cancer, indicating that the higher level of physical activity, the lower risks of colorectal cancer, and this result is supported by many other studies including Simons et al. (2013) and Des Guetz et al. (2013). Harriss et al. (2009), however, showed that physical activity did not affect the incidence of colorectal cancer. In this study, more frequent sports are correlated with higher incidence of colorectal neoplasia, which is different from the results of previous studies. The reason for this might be from different standards in estimating the frequency, duration and intensity of activities, as suggested in a review by Slattery et al. (2003). Limited number of participants might also cause this inconsistent result, hence this feature is not included in the final model generation.

## Conclusion

In this study, we developed a funnel strategy in FIT based colorectal neoplasia screening with the addition of a filtering step between FIT and colonoscopy. This filtering step was performed through a classifier model based on FIT results and a cohort of six other features using logistic regression algorithm, with a yielding of 0.92 (AUC score) in discriminating colorectal neoplasia participants from normal participants. This study will help increasing the sensitivity of FIT-based CRC screening and reducing the need of colonoscopy examination.

## Data Availability

The original contributions presented in the study are included in the article/Supplementary Material, further inquiries can be directed to the corresponding authors.
